# Functional Neurologic Symptom Disorders: Bridging the Chasm between the Psychoanalytic and Neuroscientific Understandings

**DOI:** 10.1192/j.eurpsy.2022.1213

**Published:** 2022-09-01

**Authors:** A. Deka, D. Banayan

**Affiliations:** 1 Rush University Medical Center, Department Of Psychiatry, Chicago, United States of America; 2 Rush University Medical Center, Department Of Psychiatry, CHICAGO, United States of America

**Keywords:** Conversion Disorder, functional neurologic symptom disorder, Psychoanalytic theory

## Abstract

**Introduction:**

Since before the time of “Anna O”, the functional neurologic symptom disorder (FNSD) has captivated psychiatry. While the definitive psychopathological mechanism for this phenomenon remains elusive, it is nevertheless of great value for patients and clinicians alike to develop a more nuanced understanding of FNSD. It is necessary to make an enquiry into the mechanism by bridging the psychoanalytic and neurobiological theories.

**Objectives:**

1.Elucidate psychoanalytic concepts to FNSD 2.Elucidate neuroscientific aspects of FNSD 3.Reconcile the chasm between the two concepts

**Methods:**

Comprehensive review of literature at the interface of psychoanalytic and neuroscientific theories of FNSD

**Results:**

Emerging evidence have found putative explanations to account for FNSD. Orbitofrontal cortex, anterior cingulate gyrus, dorsolateral prefrontal cortex and striatothalamocortical circuits have been implicated. Number of total studies remain small with each study having few participants. This necessitates a degree of caution in interpreting results. Thus far, mechanisms such as signal rerouting or hypoactivation of specific frontal regions appears to play a material role in FNSD. Neuroscience may be approaching to providing evidence that psychological defenses may have neurobiological correlates that can be measured in certain conditions. However, a definitive answer remains elusive.

**Conclusions:**

The expanding narrative of a relatively nascent dialogue between neuroscience and psychoanalysis remains not only clinically relevant, but also promotes a holistic view of patients with psychiatric illnesses. Through our discussion, psychoanalytic theory is woven into the current neurobiological framework for FNSD, which we believe will assist clinicians provide empathic care and help patients develop a more adaptive and meaningful explanatory paradigm of their lived experience.

**Disclosure:**

No significant relationships.

